# Frankincense and myrrh suppress inflammation via regulation of the metabolic profiling and the MAPK signaling pathway

**DOI:** 10.1038/srep13668

**Published:** 2015-09-02

**Authors:** Shulan Su, Jinao Duan, Ting Chen, Xiaochen Huang, Erxin Shang, Li Yu, Kaifeng Wei, Yue Zhu, Jianming Guo, Sheng Guo, Pei Liu, Dawei Qian, Yuping Tang

**Affiliations:** 1 Jiangsu Collaborative Innovation Center of Chinese Medicinal Resources Industrialization, National and Local Collaborative Engineering Center of Chinese Medicinal Resources Industrialization and Formulae Innovative Medicine; Nanjing University of Chinese Medicine, Nanjing 210023, PR China; 2 Jiangsu Key Laboratory for High Technology Research of TCM Formulae, Nanjing University of Chinese Medicine, Nanjing 210023, PR China; 3 Basic Medical College, Nanjing University of Chinese Medicine, Nanjing 210023, PR China

## Abstract

Frankincense and myrrh are highly effective in treatment of inflammatary diseases, but lacking of the therapy mechanisms. We undertook this stuty to evaluate the effects on Adjuvant-induced Arthritis (AIA) rats and to explore the underlying mechanisms by analyzing the metabolic profiling and signaling pathway evaluated by expression of inflammatory cytokines, *c-jun* and *c-fos* and corresponding phosphorylation levels. The results stated the elevated expression levels of TNFα, PGE_2_, IL-2, NO, and MDA in serum and swelling paw of AIA rats were significantly decreased after treatment, which exerted more remarkable inhibitive effects of combined therapy. The metbolic profiling of plasma and urine were clearly improved and twenty-one potential biomarkers were identified. Moreover, the inhibited effects of five bioactive components on cytokine transcription in PHA stimulated-PBMC showed the MAPK pathway might account for this phenomenon with considerable reduction in phosphorylated forms of all the three MAPK (ERK1/2, p38 and JNK) and down regulation of *c-jun* and *c-fos.*

Rheumatoid arthritis (RA) is a globally prevalent chronic autoimmune inflammatory disease characterized by a hyperplastic synovial membrane, which can destroy adjacent articular cartilage and bone in joints^1^,^2^,^3^. Pathologic features of the affected joints include hyperplasia of synovial tissue composed of proliferating synoviocytes and infiltrating leukocytes, including T cells and B cells, which are likely activated by autoantigens^4^.

Inflammatory cytokines play pivotal role in the pathophysiology of RA. The activated leukocytes and synovial fibroblasts in the joint tissue secrete several proinflammatory mediators such as TNF-*α*, IL-1, IL-6, IL-8, PGE_2_, INF-*γ* etc. to cause inflammation and joint degradation^5^. Both the autoreactive T and B cells play essential roles in the autoimmune responses which cause tissue inflammation, autoantibody production and clinical onset of RA^6^,^7^,^8^. T cell-derived cytokines, including IL-1, IL-2, TNF-*α*, IFN-*γ*, LT, IL-3, and IL-6, exert important and distinct function in RA pathogenesis^9^,^10^. Elevated serum levels of both PGE_2_ and leukotriene B_4_ (LTB_4_) have been reported to correlate with the severity of RA^11^. Besides, reactive nitrogen species (RNS), such as nitric oxide (NO) have been involved in the development of joint destruction in RA^12^. However, the concrete pathogenesis of RA is still unclear completely, even though viral infection and genetic predisposition might be the possible reasons^6^,^13^.

The extent of metabolic changes and types of metabolites could be applied as good markers of cytokines-mediated inflammatory processes in RA. A novel system approach to assess metabolic changes in disease is metabolomics^14^, which indicates the overall physiological status coresponding to pathological stimuli, such as genetic, environmental, or lifestyle factors^15^. Changes of metabolites with low molecular weight often mirror the end result of genomic and protein perturbations in disease, and they are closely associated with phenotypic changes. Besides, the pathogenesis of diseases and the action mechanisms of therapy could also be elucidated by identifying biomarkers, analyzing metabolic pathway, discovering drug- target interactions, and so on. Therefore, metabolic profiling has attracted an interest for investigating the RA disease and evaluating therapeutic effects of drugs^16^,^17^,^18^.

Currently, the first-line therapies for RA are focused on alleviation of inflammation, pain and joint damage by using glucocorticoids^19^, disease-modifying antirheumatic drugs (DMARDs)^20^ and non-steroidal anti-inflammatory drugs (NSAIDs)^21^,^22^. But they are limited by a number of well-characterized clinical side-effects, such as cushing syndrome^23^,^24^ and diabetes^25^ for glucocorticoids, hepatotoxicity, blooddyscrasias, and interstitiallung disease for DMARDs^26^,^27^, and gastrointestinal^28^ and cardiac toxiceffects^29^ for NSAIDs. Though the use of TNF-*α* and IL-1*β* antagonist have shown substantial efficacy, high cost of medications, and hypersensitivity to infections^30^ also can not be ignored. Consequently, there has been a great demand for new antirheumatic agents caplable of acting on multiple cytokines or mediators of inflammation, with fewer toxic or side-effects. Fortunately, effective treatments for rheumatism were available from practitioners of traditional Chinese medicine. Therefore, many researchers have aimed at developing potent therapies and drugs from Chinese medicines with fewer side-effects on RA patients.

Ruxiang (Frankincense) is dried gum resin of *Boswellia carterii* or one of 43 species in the genus Boswellia of the family Burseraceae. It has been commonly used to reduce swelling and alleviate the pain of inflammatory diseases or tumors^31^,^32^, and to invigorate the circulation of blood in China and as an antiarthritic in Ayuredic medicine in India for thousands of years^31^. Moreover, it is also used as dietary supplements for patients with arthritis or other inflammation and pain related disorders in USA^33^. Previous studies *in vitro* have shown that the boswellic acids isolated from Frankincense exhibited potential immunomodulatory effects^34^,^35^.

Myrrh, as a traditional natural medicine, is an aromatic gum resin, which was the plant stem resinous exudate of *Commiphora myrrha* (Nees) Engl. (Burseraceae) and various other different species of Commiphora family. It has many medicinal powers and has been used widely in clinic for treatment of pain and inflammatory diseases, such as stomach complaints, skin infections, ache, dysmenorrhea, chest ailments, and so on, in India, China, Rome, and Greece^36^,^37^,^38^. Especially, the myrrh was a common analgesic and has been used to clean wounds and sores for more than 2000 years, until the European discovered the morphine. Pharmacological studies also have showed that myrrh possesses multiple activities, including anti-inflammatory, cytotoxic, anesthetic, and antimicrobial effects^39^.

In China, Frankincense and Myrrh are often used together on clinic in order to obtain a synergistic effect for relieving pain and activating blood circulation, and especially to treat inflammatory diseases (e.g., RA). However, the evaluation of the effects and mechanisms are lack.

So, in this study, the adjuvant-induced arthritis (AIA) as immune-mediated rat model was used to assess the anti-arthritic efficacy of individual and combined extracts of Frankincense and Myrrh resin. The levels of TNF-*α*, and IL-2, PGE_2_, NO, and MDA were determined. To elucidate the mechanism, the metabolic changes in plasma and urine from AIA rats based on UPLC/Q-TOFMS were investigated. The potential biomarkers and metabolic pathway were also identified. Furthermore, the actions of five bioactive compounds (chemical structures see Fig. 1) drived from Frankincense (compounds **1**, **4**, and **5**) and Myrrh (compounds **2** and **3**), respectively, were investigated on TNF*α*, IL-1*β*, IL-2, IL-10, IL-12, INFγ, and *c-jun* and *c-fos* expression in PHA stimulated-PBMC (peripheral blood mononuclear cell) to explore the possible signaling pathways. These data would be useful to further develop and improve the anti-inflammatory agents.

## Results

### Rat hind paw volume

At beginning of the experiment, i.e. day 0, no significant differences were found in rat hind paw volume (HPV) among all the groups (*P* > 0.05). A significant increase in HPV was observed for the adjuvant injected group on day 13, 20, 24, 27 and 30 day compared to the healthy control rats (P < 0.001). Meanwhile, the paw edema volume was significantly reduced in group treated with standard drug of indomethacin (IMT), individual extract of Frankincense or Myrrh, and combined extracts treated groups which showed significant difference when compared with the arthritis group (P < 0.001 or P < 0.01 or P < 0.05) (See Table 1).

### Cytokine analysis

On day 30 after adjuvant inoculation, levels of TNF*α*, PGE_2_, IL-2, NO, and MDA in serum were significantly increased (*p* < 0.001) in model rats (group II) than that of control group (group I). After treated with individual extract of Myrrh (46.15 mg/kg·d, 76.92 mg/kg·d p.o., respectively) or Frankincense (33.67 mg/kg·d, 56.12 mg/kg·d, p.o., respectively) and combined extracts (54.28 mg/kg·d, 90.48 mg/kg·d, p.o., respectively), all of cytokines determined in this test were significantly decreased (*P* < 0.001 or *P* < 0.01) (see Table 2). What was interesting was the effects of the combined extract at dosage of 90.48 mg/Kg·d and extract of Frankincense extract at dose of 56.12 mg/kg·d exerted remarkablely similar with that of IMT (10 mg/kg·d). The levels of TNF-*α*, PGE_2_, IL-2, NO, and MDA in swelling paw tissue had same trends in model rats than that of control group, and therapeutic trends by test drugs (see Table 2).

From the pathologic changes of hind paw and the regulation of pre-inflammation cytokines by the extract of Frankincense and combined extract, it was showed that they slowed the progression of inflammation obviously, accelerated bone resorption, prevented periosteal bone proliferation and cartilage destruction.

### UPLC/Q-TOF/MS analysis of metabolic profiling

**Figure 2:**
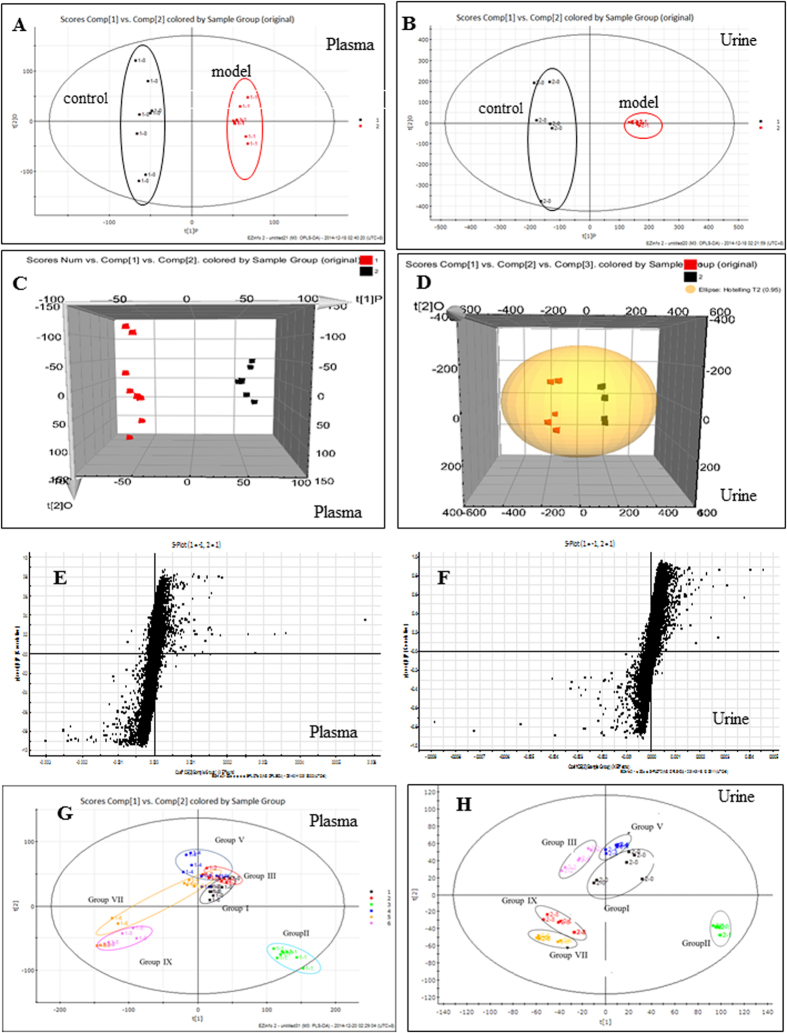
PCA model results between AIA rats and controls in negative mode. ((**A**) 2-D plot of plasma; (**B**) 2-D plot of urine). 3D PLS-DA scores plot of LC–MS spectral data ((**C**) plasma, R2 = 0.87, Q2 = 0.92; (**D**) urine, R2 = 0.93, Q2 = 0.88). S-plot of OPLS-DA model for AIA vs control group. ((**E**) plasma, R2 = 0.86, Q2 = 0.81; (**F**) urine, R2 = 0.96, Q2 = 0.84). PCA analytical results from AIA rats treated with drugs in different groups at negative mode. ((**G**) For plasma; (**H**) for urine).

Typical based peak intensity (BPI) chromatograms of plasma and urine samples, collected from model rats and normal rats in negative modes were shown in Figure S1A-B. The unsupervised PCA model was used to separate plasma or urine sample into two blocks between model rats and normal rats. A total of 252 ions in plasma samples and 347 ions in urine samples at negative modes were detected from model rats and normal rats. PCA scores plots showed clear clustering of them (Fig. 2A–D). The supervised OPLS-DA divided samples into two blocks and distinguished model rats from normal rats’ cohorts with 100% sensitivity and no less than 95% specificity using a leave one out algorithm, which indicated that the OPLS-DA model was reliable. From the loading plots of OPLS-DA, 36 ions in plasma samples and 43 ions in urine samples at negative modes were deemed discriminatory (*p* < 0.05), and identified as being responsible for the separation between model rats and normal rats (Fig. 2E,F).

### Identification of potential biomarkers and metabolic pathway function analysis

In the plasma, ten endogenous metabolites, contributing to the separation between the groups, were identified (Table 3). The precise molecular mass was determined within measurement errors (<5 ppm) by Q-TOF/MS/MS. Compared with normal rats, alanyl tryptophan, linoelaidic acid, and docosahexaenoic acid were up regulated (*p* < 0.05), while LysoPC(17:0), LysoPC(15:0), LysoPE(20:2(11Z,14Z)/0:0), LysoPE(20:1(11Z)/0:0), LysoPE(18:1 (9Z)/0:0), LysoPC(18:2(9Z,12Z)), and LysoPC(16:0/0:0) were down regulated significantly (*p* < 0.05).

In the urine, detected significant variables in the negative ion mode were summarized in Table 3. Eleven endogenous metabolites were tentatively identified by the methods described above. The metabolite of indoxyl sulfate was observed to be up regulated significantly (*p* < 0.05), whereas the other metabolites of 4,6-dihydro xyquinoline, malonyl carnitine, bicine, homocysteine thiolactone, citric acid, isoval eryglutamic acid, glucaric acid, 9′-carboxy-gama-tocotrienol, topiramate, and xanthosine were down regulated obviously (*p* < 0.05). These differences in plasma and urine might denote the potential targeted biomarkers for differentiating RA pathological from normal states.

The metabolic pathway analysis with MetPA revealed that the identified biomarkers were critical for the host responding to RA. Five metabolic pathways were disturbed, which included glycerophospholipid metabolism, citrate-cycle- (TCA-cycle), glyoxylate and dicarboxylate metabolism, ascorbate and aldarate metabolism and glycosylphosphatidylinositol (GPI)-anchor biosynthesis. The pathway impact value calculated from pathway topology analysis above 0.1 was filtered out as potential target pathway.

### Intervention effects of Frankincense, Myrrh and combined extracts

In order to elucidate the intervention efficacy of Frankincense, Myrrh, and combined extracts, PCA analysis was carried out to obtain the changes among group I-IX. The variations of plasma and urine metabolic profiling of Frankincense, Myrrh and their combination-treated rats was restored back to the levels more than control-like on the 30^th^ day (Fig. 2G,H). Furthermore, ten endogenous metabolites in plasma and eleven endogenous metabolites in urine were significantly affected by Frankincense and Myrrh combined extracts (*p* < 0.05 or *p* < 0.01), while the IMT regulated eleven endogenous metabolites in urine including topiramate level except for the same metabolites with ones regulated by combined extracts. All of these metabolites were restored back to a control-like level and there was no significant difference between the IMT and combined extract - treated group (*p* > 0.05). There was no obvious effects on metabolites levels of LysoPC(17:0), LysoPC(15:0), and LysoPC(16:0/0:0) (in plasma) for Frankincense extract. The levels of LysoPC(17:0), LysoPC(15:0), and LysoPE(18:1(9Z)/0:0) were also not regulated by Myrrh extract. It was interesting that the metabolites levels of citric acid, isoval eryglutamic acid, glucaric acid, 9′-carboxy-gama-tocotrienol, and xanthosine in urine were neither affected by frankincense extract nor myrrh extract, but by their combination.

These changes may not immediately in response to therapeutic effects of Frankincense and Myrrh for the AIA rats, but they were generated from perturbation in organism by administration of Frankincense and Myrrh. The contents of the potential biomarkers in Table 3 were considered as biomarkers for effect of treatment.

### Dose optimization of five bioactive compounds

To evaluate bioactivitives of five bioactive compounds including 3-hydroxylanosta-8,24-dien -21-oic-acid (**1**), 2-Methoxy-5-acetoxy-fruranogermacr -1(10)-en- 6–one (**2**), abietic acid (**3**), elemonic acid (**4**), and Acetyl elemolic acid (**5**), a series of dosages were applied on PBMC and MTT assays were carried out (Table S1). Then, dosages of 70, 1.0, 1.0, 4.0 and 2.3 *μ*g/ml were selected for compound **1**, **2**, **3**, **4** and **5** respectively for further analysis on different targets.

### Effects of five compounds on cytokine transcription in PHA stimulated-PBMC

**Figure 3:**
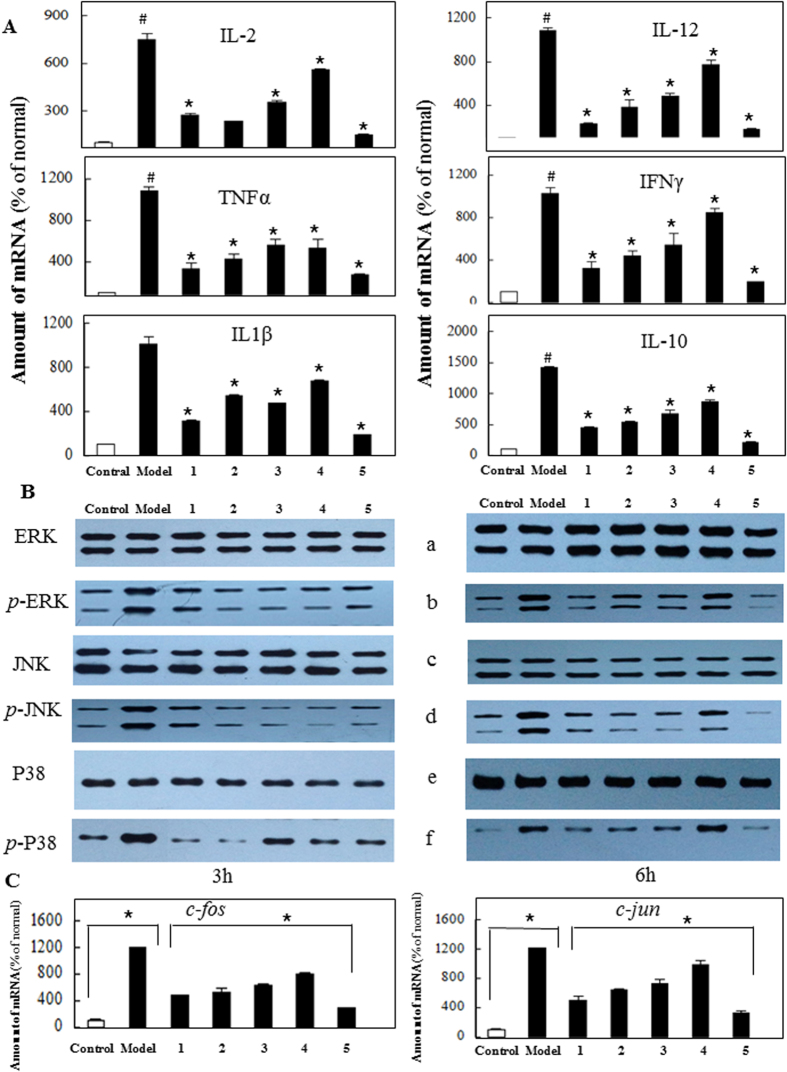
(**A**) Analysis of compounds **1–5** on PHA induced proinflammatory cytokine expression in PBMC. PBMCs were induced with PHA (10 ng/ml) for 6 hours and inhibitory effect of them on cytokines expression was studied by RT-PCR analysis. (**B**) Analysis of compounds 1–5 on MAPK. (a) non-phospho ERK, (b) Phospho-ERK, (c) non-phospho JNK, (d) Phospho-JNK, (e) non-phospho p38, (f) Phospho-p38. By Western blotting, the inhibitory effects of them on active forms of MAP kinases were analysed in PHA stimulated PBMC, using antibodies recognizing the phosphorylated and non-phophorylated forms of ERK1/2, JNK and p38 MAPK. (**C**) Analysis of compounds 1–5 on PHA induced *c-fos* and *c-jun* expression in PBMC. PBMCs were induced with PHA (10 ng/ml) for 6 hours and inhibitory effect of the five compounds on *c-fos* and c-jun cytokines expression was studied.

The results showed that the transcription levels of IL-1*β*, IL-2, IL-10, IL-12, TNF*α*, INF*γ* could be enhanced in PHA stimulated-PBMC while this tendency could be decreased by treantment of compounds **1–5** (Fig. 3A). Especially, compound **5** showed the most marked inhibiting effect for all tested cytokines.

### Kinase phosphorylation of MAPK signaling pathway on PBMC treated with five compounds

Mitogen activated protein kinases (MAPK) pathway is a major pathway accounting for immune responses, including the regulation of cytokine responses, and chemokine responses. PBMC were stimulated with PHA for 3 and 6 h to activate MAPK signaling pathway. The phosphorylation levels of critical kinases, including ERK, JNK and the p38, in PBMC treated by agonist and tested compounds, were assessed by Western blotting (Fig. 3B). The phosphorylation levels of ERK, JNK and p38 were significantly enhanced by treatment of PHA, the agonist of MAPK pathway while decreased by treatment of five compounds (Fig. 3B).

### Down regulation of *c-jun* and *c-fos*

PBMCs treated with five compounds showed marked inhibition of both *c-jun* and *c-fos* expression (Fig. 3C). The marked reduction in *c-jun* levels could affect AP-1 levels and thereby the downstream signals resulting in inhibition of inflammatory cytokines.

## Discussion

Freund complete adjuvant (FCA)-induced secondary inflammation mimics sub-acute RA^40^. AIA rat model is a useful tool to study the pathology of RA, due to its similarity with human disease sharing common signs and symptoms^41^,^42^. In AIA rat model, swelling of hind paws, increased levels of inflammatory cytokines were indicators of inflammation reaction for immune arthritis. Immunization of SD rats with Mtb not only induces inflammation, but also primes and expands T cells directed against mycobacterial antigens^40^. Activation of T cells following Mtb injection involves the processing and presentation of mycobacterial antigens to specific T cells and subsequent clonal proliferation of the activated T cells. In our study, Frankincense and myrrh, especially their combined extract significantly suppressed arthritis progression as evidenced by reduction of preinflammatory factors. The beneficial effect of frankincense and myrrh on inflammation was previously reported^43^,^44^,^45^,^46^,^35^. Acetone extract of *Boswellia carterii* gum resin decreased arthritic scores, reduced paw edema and suppressed local tissue TNF-*α* and IL-1*β* in Lewis rats significantly^32^. It is worth mentioning that the combination of Frankincense and Myrrh was more effective in suppressing the intensity of joint inflammation.

Furthermore, five bioactive compounds derived from frankincense and myrrh could inhibit the expression of IL-1*β*, IL-2, IL-10, IL-12, TNF*α*, INF*γ*, *c-jun* and *c-fos* in PHA activated PBMC. The phosphorylation levels of ERK, JNK and p38 were significantly inhibited in PHA activated PMBC by compounds **1–5**. Especially, the compound **5** showed a more prominent effect against pre-inflammatory factors. Current data could elucidate that five bioactive compounds could exert anti-inflammatory effects via blocking MAPK pathway.

Nitric oxide (NO) is a critical biochemical mediator of inflammation and involved in autoimmune mediated tissue damage and inflammation^47^,^48^,^49^. In our study, nitricoxide levels were increased in untreated adjuvant arthritis rats. And it arised from the possibility that excessive nitricoxide production by inducible nitricoxide synthase (iNOS) induced by TNF-*α* and IL-1 and resulted in the formation of excessive amounts of superoxide (O2−)^50^, which reacted with nitricoxide (NO·) to generate peroxynitrite (ONOO−). It had been reported that peroxynitritere acting with tyrosine residues of protein stoproduce nitrotyrosine contributed to rheumatoid arthritis pathogenesis^51^. However, determination of serum nitrotyrosine might provide an evidence for this proposed action mechanism. The decrease in serum nitricoxide levels by Frankincense and Myrrh might be attributed to its inhibition of reactive oxygen species production in the synoviocytes through modulation of TNF-*α* and IL-1 synthesis.

The pre-inflammation cytokines of TNF-*α* and IL-1 could promote the release of PGs (e.g., PGE_2_ causes synovial inflammation), leukotrienes, and oxygen free radical and generate collagenases and neutral protease, which induced the cartilage matrix breakdown, cartilage resorption and bone destruction^52^. MDA was a peroxidation product produced because of lipid attacked by free radicals and the level of MDA represented the intensity of body injury. In our study, Frankincense and Myrrh combined therapy exhibited better effecacy than Frankincense and Myrrh alone for inhibiting the PGE_2_ and MDA levels except for TNF-*α*. This enhanced effect mechanisms between Frankincense and Myrrh still need further to be investigated.

The results of plasma metabolomics study of AIA rats stated that endogenous metabolites of LysoPCs and LysoPEs levels were decreased, which led to the metabolic disorder of phospholipid metabolism in inflammation. The decreased levels of glycerophospholipid metabolites, including LysoPC(17:0), LysoPC(15:0), LysoPE(20:2(11Z,14Z)/0:0), LysoPE(20:1(11Z)/0:0), LysoPE(18:1 (9Z)/0:0), LysoPC(18:2(9Z,12Z)), and LysoPC(16:0/0:0), indicated a marked perturbation in the phospholipid metabolic pathways in AIA. Lyso-PC, an important component of oxidized low-density lipoprotein (oxLDL), has been confirmed to be a chemoattractant for T lymphocytes^53^. Lyso-PC also induces antibody formation and macrophage stimulation; therefore, Lyso-PC levels can impact on the inflammation state of an organism. Thus, it can be speculated that the abnormal plasma levels of Lyso-PC in AIA rats increase the progression of AIA. Other differential metabolites, such as Lyso-PE, are important phospholipid synthetic pathway intermediates; abnormalities in these metabolites also reflect the impact of AIA on glycerophospholipid metabolic pathway.

The biosynthesis of unsaturated fatty acids metabolic disturbance induced the elevated levels of linoelaidic acid and docosahexaenoic acid, which was metabolized to PGs, thromboxane (TXs) and leukot rienes (LTs) through lipoxygenase and cyclooxygenase pathway and thus regulated inflammation. PGE_2_ (prostaglandin E_2_) is generated from AA via the COX pathway, and it is an important mediator of inflammation, pain, and joint destruction and is found in AIA rats in the synovial.

By urine metabolomics research, the glyoxylate and dicarboxylate metabolism, citrate cycle (TCA cycle), and glycosylphosphatidylinositol(GPI)-anchor biosynthesis were disturbed and identified the decreased metabolites. Citric acid is an important intermediate of the tricarboxylic acid (TCA) cycle taken place in mitochondria. TCA cycle is one of the most important energy metabolism. Urinary level of citric acid is used as diagnosis of kidney stones, renal tubular acidosis and bone diseases^54^. The decreased level of urinary citric acid may suggest that the impaired action of citrate synthase in TCA cycle in AIA rats, and probably due to perturbed metabolism in cartilage and chondrocytes. Frankincense, Myrrh and combined extracts could recover this downward trend of citric acid level. Trans fatty acids (TFA) are reported to contribute to inflammation and coronary heart disease. The remarkably increased linoelaidic acid implied the effects on the progress of inflammation degree.

4,6-dihydroxyquinoline is downstream metabolite of tryptophan through kynurenine pathway. Tryptophan, an essential amino acid, plays a fundamental role in physiology and biochemistry. Tryptophan metabolism through the kynurenine pathway was considered as one of many mechanisms involved in how immune system continuously modulated the balance between responsiveness to pathogens and tolerance to non-harmful antigens^55^. The level of 4,6-dihydroxyquinoline is significant decreased in urine, and the high level of alanyl tryptophan in plasma, suggested the unbalanced immune response. The regulation to health condition of 4,6-dihydroxyquinoline and alanyl tryptophan level may imply that kynurenine pathway was regulated after the treatment of AIA rats with Frankincense, Myrrh and combined extracts.

Indoles endogenous metabolites are usually produced through tryptophan metabolism. Indoxyl sulfate (IS) is metabolized by the liver from indole, which is toxic and produced from tryptophan by intestinal flora. The excretion of IS increased significantly in AIA rats. After 17 days’ therapeutic intervention with Frankincense, Myrrh and combined extracts, the excretion of it went down. These change trends indicated that therapeutic effects of them might base on the regulation of the dysfunction of tryptophan metabolism.

**Figure 4:**
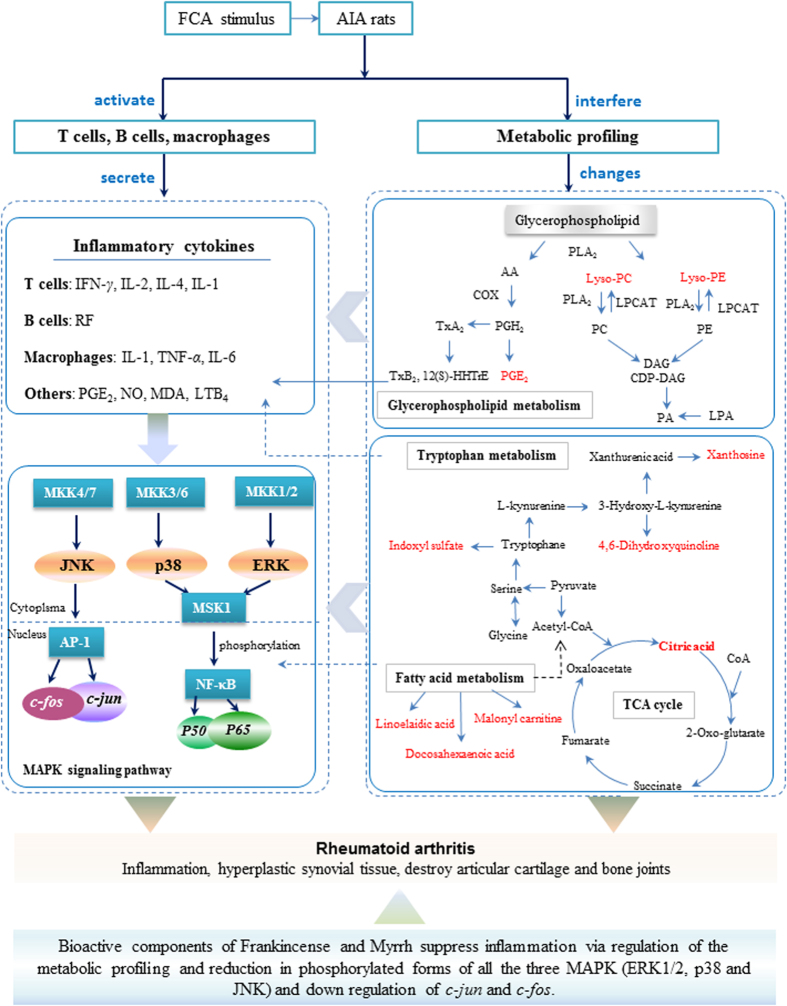
Correlation networks of main potential biomarkers in response to AIA and the effects of treatment for AIA. The metabolites marked in red denote the identified potential biomarkers.

Considering the potential linkages, the correlation networks of the potential biomarkers in response to effect of treatment for AIA rats is described in Fig. 4. We speculated how biomarkers either up- or down-regulated implicated in inflammatory and immune responses through the metabolic pathway and literature search.

In conclusion, the present studies suggested that combined Frankincense and Myrrh exerted a significant protective effect on HPV, inflammatory cytokines, as well as cytokines expression level. Thus, administration of combined Frankincense and Myrrh suppressed arthritic progression in rats more effective than single drug treatment. These findings might supply beneficial hints for the treatment of rheumatoid arthritis and deserves further clinical investigations.

## Materials and Methods

### Animals

Adult male Sprague-Dawley (SD) rats (200 ± 10 g) were purchased from Nanjing University of Chinese Medicine (rodent license no. SCXK 20080033) and kept at controlled environment conditions at a constant temperature (23 ± 2 °C), humidity (60 ± 10%), and a 12/12 h light/dark cycle. Rats were acclimatized for one week before any experimental procedures and were allowed standard rat chow and water ad libitum. All experimental procedures were carried out in accordance with the Guide for the Care and Use of Laboratory Animals, and before the animal experiments were carried out, the procedures were approved by the Research Ethical Committee of Nanjing University of Chinese Medicine (Nanjing, China).

### Chemicals and cytokines

Freund complete adjuvant (FCA) (100M8717 CAS9007-81-2) was purchased from Sigma-Aldrich (St. Louis, MO, USA). TNF-*α*, PGE_2_, and IL-2 enzyme linked immunosorbent assay (ELISA) kits were procured from Shanghai Westang Bio-tech CO., Ltd., Shanghai, China. Nitrite test kit (Nanjing Jiancheng Bioengineering Institute Co., Ltd., Nanjing, China, no. 20081204). Sodium citrate (No. 050580052) and Indomethacin (No. H31020148) were obtained from Shanghai Chemical Reagent Company, LTD.

Frankincense and myrrh were collected from Guangdong, China, in April 2013 and traded in the market as medicinal resin. They were identified as resin derived from *Commiphora myrrha* (Nees) Engl. and *Boswellia carterii* Birdw., respectively, by the corresponding author. The voucher specimens (nos. NJUTCM130123 and NJUTCM130134) were deposited at the Herbarium of the Nanjing University of Chinese Medicine.

### Induction of arthritis rats model and treatment protocol

To induce arthritis, the right hind footpads of male SD rats were sterilized with 70% alcohol and were intradermally injected with 100 *μ*L FCA (10 mg/ml) suspension of heat-killed Mycobacterium tuberculosis in vehicle (Sigma Aldrich Co-USA) according to the method by literatures^56^,^57^. Control animals were injected intradermally with saline in equal volume. Then the hind paw volumes were measured as the parameter of paw swelling for a period of 13 or 30 days. Chronic inflammation was allowed to progress for 12 days then rats were divided into 9 groups of eight rats each. Urine (in 12 hour intervals) and blood samples were collected on the 0, and 31^th^ day.

We did our best in order to minimize the suffering of experimental animals and to use the number of rats which enables us to generate reliable data. Seventy two rats were randomly allocated into nine groups. Group I (n = 8) served as normal rats which received an equal volume of vehicle control. Group II (n = 8) was arthritic model control treated with vehicle only. Group III (n = 8) was arthritic control which received the indomethacin (IMT) (10 mg/Kg·d p.o.). Groups IV (n = 8) and V (n = 8) were treated with combined extracts of Frankincense and Myrrh (54.28 mg/Kg·d, 90.48 mg/Kg·d, p.o., respectively). Groups VI (n = 8) and VII (n = 8) were treated with extracts of Frankincense (33.67 mg/Kg·d, 56.12 mg/Kg·d, p.o., respectively). Groups VIII (n = 8) and IX (n = 8) were treated with extracts of Myrrh (46.15 mg/Kg·d, 76.92 mg/Kg·d p.o., respectively). The drugs were orally administered through feeding tube daily in 0.02% Tween-80 vehicle for 17 days starting 2 h before injecting FCA on day 13 and were continued up to day 30.

### Sample collection and preparation

The rats were fixed in supine position and anesthetized with 10% chloral hydrate by intraperitoneal injection, blood samples were collected in heparinized tubes on the 31^th^ day from carotid artery. They were then anti-coagulated in 3.8% natrium citricum (9:1), centrifuged at 3 000 r/min for 10 min and the supernatants were stored at −20 °C until detection of TNF_*α*_, NO, MDA, PGE_2_, IL-2. Then the animals were sacrificed by cervical dislocation. The hind paw were separated and immersed in physiological saline (tissue : saline, 9:1, g/v) for 24 h, centrifuged at 3 000 × *g* for 10 min, collected supernatant, and stored at −20 °C until detection of TNF_*α*_, NO, MDA, PGE_2_, IL-2. Urine samples were collected in 12 hour intervals, then centrifuged at 13 000×*g* for 10 min and stored at −20 °C until analysis.

Two hundred microliters of plasma was added to 600 *μ*L of acetonitrile, and this mixture was vortexed for 30 s and centrifuged at 13 000 *g* for 10 min to obtain the supernatant. Prior to analysis, the urine samples were thawed at room temperature and centrifuged at 13 000×*g* for 10 min. The supernatant liquid (1 mL) was added to 3 mL of acetonitrile, vortex mixed for 30s, and centrifuged at 13000×*g* for 10 min to obtain the supernatant. The plasma and urine supernatants were removed and evaporated to dryness in a 40 °C water bath under a gentle stream of nitrogen. The residues were reconstituted in 200 *μ*L mobile phase of 70% acetonitrile-water solution, centrifuged at 13 000 × *g* for 5 min and filtered through a 0.22 *μ*m membrane filter. The filtrates were transferred to an auto-sampler vial and stored at 4 °C. A 5 *μ*L aliquot of each plasma or urine sample was injected for LC/MS analysis.

### UPLC–QTOF/MS and UPLC-QqQ/MS analysis

Chromatography was performed on an AcQuity^TM^ UHPLC system (Waters Corp., Milford, MA, USA) with a conditioned auto-sampler at 4 °C. The separation conditions and mass spectrometric detection methods are listed in Text S1.

### Hind paw volume measurement

The hind paw volume (HPV) of all animal groups was measured by water displacement plethysmometer^58^ at 0, 13, 20, 24, 27 and 30^th^ day after the injection of FCA emulsion. The inhibited effects of Frankincense and Myrrh were determined by comparing the changes in volumes of hind paws and expressed in milliliter (mL ± SD).

### BCA protein assay in rat tissues

The BCA protein assay kit was obtained from Westang Bio-tech Co., LTD (Shanghai, China). Test protocols were listed in Text S1.

### Determination of proinflammatory cytokines (IL-2, TNF-*α*, and PGE_2_)

Serum interleukin 2 (IL-2), tumor necrosis factor-α (TNF-*α*) and PGE_2_ levels were determined by avidin biotin peroxidase complex enzyme-linked immunosorbent assay (ABC-ELISA) kits. All of the ELISA test kits were used according to the manufacturers’ instructions. The procedure were discribed in detail in Text S1.

### Nitric oxide (NO) determination

NO was measured through the nitric oxide assay kit (nitrate reductase) following the instruction of the manufacturer. Nitric oxide is chemically active and can be rapidly oxidized to nitrite (NO_2_^−^) and nitrate (NO_3_^−^) *in vivo*. Nitrite can be further oxidized to nitrate. In this study, NO was determined by colorimetric method based on the nitrate to nitrite conversion through nitrate reductase. Samples preparation and test protocols were shown in Text S1.

### Malondialdehyde (MDA) assay

MDA was measured through the MDA kit following the instruction of the manufacturer. MDA is a product of lipid peroxidation and degradation, which can react with thiobarbituric acid (TBA) to form a red product for colorimetric assay (532 nm). Prepare the reagent as guidance of the kit. Obtain the serum and supernatant of tissue described above. Sample solutions and determination discribed in Text S1.

### Metabolomic data processing and multivariate analysis

UPLC/MS data were detected and noise-reduced in both the UPLC and MS domains such that only true analytical peaks were selected for further processing by the software and according to the method discribed in our previous study^59^. The details are listed in Text S1.

### Biomarker identification and metabolic pathway analysis

The identities of the potential biomarkers were confirmed by comparing their mass spectra and chromatographic retention times with the available reference standards and a full spectral library containing MS/MS data obtained in the positive and/or negative ion modes. The Mass Fragment application manager (Waters MassLynx v4.1, Waters corp., Milford, USA) was used to facilitate the MS/MS fragment ion analysis through the use of chemically intelligent peak-matching algorithms. This information was then used to search multiple databases and analyzed the potential metabolic pathway using MetPA (See Text S1). Potential biological roles were evaluated by an enrichment analysis using MetaboAnalyst.

### Isolation of peripheral blood mononuclear cells (PBMC)

Peripheral blood was taken from healthy volunteers. Mononuclear cells were isolated in a Ficoll–Hypaque (Pharmacia, Piscataway, NJ) density gradient using standard procedures. The buffy coat containing PBMCs was removed carefully following centrifugation and washed twice in RPMI 1640 medium containing 10% FCS (Sigma). Cells were counted and assessed for viability.

### Real-time quantitative PCR

Total RNA was isolated from treated PBMCs using Trizol reagent (Sigma, St Louis, MO, USA) following the protocol provided by the manufacturer. Real-time quantitative PCR was performed by using SYBR Green Master mix and Rox reference dye, according to the manufacturer’s instructions. The cDNAs were obtained from the reverse transcription of the RNA from rat brain tissues and astrocyte cells. The primers were listed below. SYBR green signal was detected by Mx3000ptm multiplex quantitative PCR machine. Transcript levels were quantified by using the ΔΔCt value method^60^. Calculation was done by using the Ct value of GAPDH to normalize the Ct value of target gene in each sample to obtain the ΔΔCt value, which then used to compare among different samples. PCR products were analyzed by gel electrophoresis on a 1.5% agarose gel, and the specificity of amplification was confirmed by the melting curves.

### Western blot analysis

PBMCs were treated with the optimized doses of compounds **1–5** for the required time points. The cells were lysed with extraction buffer (20 mM HEPES, 150 mM NaCl, 1% Triton X-100, 1 mM EDTA, 1 mM PMSF, 10 *μ*g/mL leupeptin, and 10 *μ*g/mL aprotinin). After 30 min at 4 °C, debris was eliminated by centrifugation at 14, 000 rpm for 20 min, and the supernatant was collected. Cell lysates were separated by 10% SDS-PAGE, transferred to polyvinylidene difluoride membranes, and blocked with 0.05% Tween 20 and 5% BSA overnight. The immunoblots were incubated with anti-phospho-specific extracellular signal-regulated kinase (ERK) Ab, anti-ERK Ab, anti-phospho-specific p38 Ab, anti-p38 rabbit Ab, anti-phospho-specific JNK rabbit Ab, or anti- JNK rabbit polyclonal Ab in PBS with 1% BSA for 1 h. Subsequently, the immunoblots were incubated with secondary antibody conjugated with HRP in 1% BSA in PBS, 0.1% Tween 20. After 1 h incubation at room temperature the bands are detected using chromogenic substrate.

### Statistical analysis

Statistical analysis Data are expressed as mean ± SEM, and statistical comparisons were carried out using one-way analysis of variance (ANOVA), followed by Student’s *t*-test. All quantitative data analyses were performed using the SPSS 11.5 software package for Windows. The results were expressed as the mean ± SD. *P* values less than 0.05 were considered significant.

## Additional Information

**How to cite this article**: Su, S. *et al.* Frankincense and myrrh suppress inflammation via regulation of the metabolic profiling and the MAPK signaling pathway. *Sci. Rep.* **5**, 13668; doi: 10.1038/srep13668 (2015).

## Supplementary Material

Supplementary Information

## Figures and Tables

**Figure 1:**
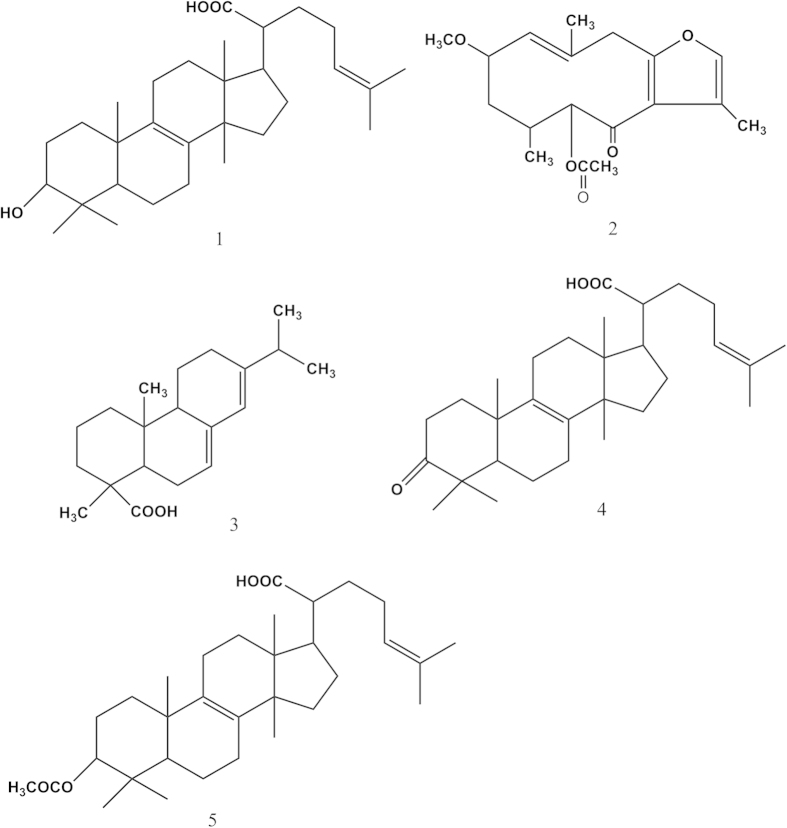
The chemical structures of five bioactive compounds drived from Frankincense (compounds 1, 4, and 5) and Myrrh (compounds 2 and 3) (1. 3-hydroxylanosta-8,24-dien-21-oic-acid; 2. 2-Methoxy-5-acetoxy-fruranogermacr -1(10)-en-6-one; 3. abietic acid; 4. elemonic acid; 5. Acetyl elemolic acid).

**Table 1:** Effects of extracts of *C. myrrha*, *B. carterii.* and their combination on rat hind paw swelling in adjuvant-induced arthritis model.

Groups	Dosage(mg/Kg·d p.o.)	Rat hind paw volume in ml ± SD (% inhibition)					
		Day 0	Day 13	Day 20	Day 24	Day 27	Day 30
Group-I	–	0.86 ± 0.12	1.03 ± 0.10	1.04 ± 0.07	1.06 ± 0.07	1.16 ± 0.43	0.96 ± 0.15
Group-II	–	0.863 ± 0.16	1.87 ± 0.15a ***	1.86 ± 0.13a ***	1.86 ± 0.12a ***	1.89 ± 0.12a***	2.00 ± 0.13a***
Group-III	10	0.83 ± 0.21	1.73 ± 0.15	1.61 ± 0.13b ***	1.54 ± 0.15b ***	1.47 ± 0.05b ***	1.38 ± 0.06b***
Group-IV	54.28	0.85 ± 0.23	1.75 ± 0.19	1.61 ± 0.12b ***	1.48 ± 0.08b ***	1.42 ± 0.05b ***	1.38 ± 0.05b ***
Group-V	90.48	0.80 ± 0.20	1.70 ± 0.15	1.64 ± 0.12b **	1.58 ± 0.11b **	1.46 ± 0.12b ***	1.36 ± 0.074b ***
Group-VI	33.67	0.82 ± 0.21	1.78 ± 0.16	1.67 ± 0.08b **	1.55 ± 0.15b ***	1.49 ± 0.11b ***	1.47 ± 0.13b ***
Group-VII	56.12	0.86 ± 0.18	1.88 ± 0.27	1.79 ± 0.20	1.56 ± 0.27b ***	1.49 ± 0.20b ***	1.39 ± 0.13b ***
Group-VIII	46.15	0.87 ± 0.16	1.87 ± 0.17	1.78 ± 013	1.63 ± 0.23b **	1.53 ± 0.14b ***	1.48 ± 0.09b ***
Group-IX	76.92	0.85 ± 0.15	1.78 ± 0.19	1.69 ± 0.11b *	1.53 ± 0.15b ***	1.43 ± 0.18b ***	1.36 ± 0.14b ***

Values are expressed as mean ± S.D., n = 8 animals in each group. Figures in parenthesis indicate percent inhibition of rats paw volume of FCA and drug treated groups Vs healthycontrol Group. Comparisons were made between: a-Group I vs GroupII- IX. B-Group II vs Group III–IX. (*P < 0.05, **P < 0.01, ***P < 0.001).

**Table 2:** Effects of Frankincense, Myrrh and combined extracts on the cytokines (NO, MDA, IL-2, PGE_2_, TNF_*α*_) levels in rats’serum and right paw swelling of adjuvant-induced arthritia model (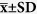, n = 8).

Groups	Dosagemg/Kg·d	NO (*μ*mol/L)		MDA (nmol/Ml)		IL-2 (pg/mL)		PGE_2_(pg/mL)		TNF_α_(pg/mL)	
		In serum	Right paw tissue	In serum	Rightpawtissue	In serum	Rightpawtissue	In serum	Rightpawtissue	In serum	Rightpawtissue
Group-I	—	73.72 ± 6.34	83.24 ± 10.86	32.47 ± 3.10	0.61 ± 0.08	48.56 ± 7.93	53.41 ± 13.71	174.05 ± 14.46	84.55 ± 18.74	24.68 ± 3.87	40.49 ± 2.86
Group-II	—	153.12 ± 10.22a ***	120.08 ± 10.29a ***	41.96 ± 1.76a **	3.40 ± 0.48a ***	143.10 ± 8.06a ***	110.35 ± 11.77a ***	459.29 ± 22.86a ***	444.19 ± 10.39a ***	110.35 ± 11.78a ***	94.72 ± 25.67a ***
Group-III	10.00	94.99 ± 10.89a ***	67.67 ± 7.49a ***	33.74 ± 2.90b *	0.80 ± 0.16a ***	79.39 ± 15.33a ***	72.00 ± 25.17a ***	220.76 ± 22.15a ***	123.50 ± 15.48a ***	47.81 ± 5.39b ***	44.40 ± 10.51a ***
Group-IV	54.28	144.61 ± 25.99	78.16 ± 7.93a ***	36.38 ± 4.01	0.95 ± 0.06a ***	97.75 ± 17.33a ***	86.56 ± 10.66a **	229.56 ± 15.65a ***	303.96 ± 54.17a ***	38.95 ± 11.22a ***	49.35 ± 9.65a ***
Group-V	90.48	92.15 ± 8.39a ***	54.66 ± 5.61a ***	38.24 ± 4.83	2.47 ± 0.57a **	65.22 ± 8.33a ***	71.40 ± 15.31a ***	345.77 ± 18.36 ***	156.23 ± 18.25a ***	66.42 ± 11.01a ***	35.81 ± 7.49a ***
Group-VI	33.67	86.48 ± 13.52a ***	60.94 ± 3.98a ***	40.20 ± 7.57	2.34 ± 0.48a ***	168.42 ± 13.12a ***	97.52 ± 8.29	342.97 ± 43.92 ***	277.43 ± 25.34a ***	69.44 ± 9.41a ***	55.71 ± 4.58a ***
Group-VII	56.12	114.84 ± 13.52a **	71.18 ± 14.62a ***	38.53 ± 5.20	2.17 ± 0.48a ***	96.40 ± 10.17a ***	63.30 ± 7.61a ***	354.70 ± 34.30 ***	159.32 ± 26.11a ***	58.93 ± 7.66a ***	37.39 ± 6.11a ***
Group-VIII	46.15	121.93 ± 27.49	73.72 ± 2.52a ***	37.85 ± 4.81	1.91 ± 0.27^b ^***	88.02 ± 8.56a ***	77.11 ± 18.44a ***	350.71 ± 11.39 ***	188.96 ± 20.33a ***	60.68 ± 10.39a ***	44.63 ± 8.20b ***
Group-IX	76.92	62.38 ± 24.23a ***	46.79 ± 6.70a ***	35.11 ± 1.24b *	1.57 ± 0.30a ***	44.84 ± 11.51a ***	60.52 ± 10.68a ***	304.01 ± 22.97 ***	263.73 ± 33.99a ***	35.87 ± 7.28a ***	31.80 ± 5.98a ***

Results were expressed as mean ± standard deviation of 8 animals per group (n = 8). Comparisons were made between: (a) Group I vs GroupII–IX. (b) GroupII vs Group III–IX. Symbols represent statistical significance: *P < 0.05, **P < 0.01, ***P < 0.001.

**Table 3:** The identified and change trend of the potential biomarkers of AIA rats intervened by frankincense and myrrh.

No.	t_R_/min	[M-H]^−^*m/z*	metabolites	Contentvariance^a^(Group II)	Effects of test drugs				Pathway (KEGG)	Resources
					GroupIII	GroupV	GroupVII	GroupIX		
1	10.75	568.3625	LysoPC(17:0)	↓	↑^b^	↑^b^	–	–	Glycerophospholipid metabolism	Plasma
2	8.56	540.3321	LysoPC(15:0)	↓	↑^b^	↑^b^	–	–	Glycerophospholipid metabolism	Plasma
3	7.81	564.3312	LysoPE(20:2(11Z,14Z)/0:0)	↓	↑^b^	↑^b^	↑^b^	↑^b^	Glycerophospholipid metabolism	Plasma
4	9.04	566.3466	LysoPE(20:1(11Z)/0:0)	↓	↑^b^	↑^b^	↑^b^	↑^b^	Glycerophospholipid metabolism	Plasma
5	7.27	538.3158	LysoPE(18:1(9Z)/0:0)	↓	↑^b^	↑^b^	↑^b^	–	Glycerophospholipid metabolism	Plasma
6	6.36	312.0784	Alanyl tryptophan	↑	↓^c^	↓^c^	↓^c^	↓^c^	Tryptophan metabolism	Plasma
7	7.49	564.3321	LysoPC(18:2(9Z,12Z))	↓	↑^b^	↑^b^	↑	↓↓^c^	Glycerophospholipid metabolism	Plasma
8	13.21	279.2318	Linoelaidic acid^d^	↑	↓^c^	↓^c^	↓^c^	↓^c^	Biosynthesis of unsaturated fatty acids	Plasma
9	12.50	327.2319	Docosahexaenoic acid^d^	↑	↓^c^	↓^c^	↓^c^	↓^c^	Biosynthesis of unsaturated fatty acids	Plasma
10	9.75	554.3479	LysoPC(16:0/0:0)^d^	↓	↑^b^	↑^b^	–	↑^b^	Glycerophospholipid metabolism	Plasma
11	6.74	160.0394	4,6-Dihydro xyquinoline	↓	↑^b^	–	↑^b^	↑^b^	5-hydroxytryptophan metabolism	Urine
12	5.27	284.0589	Malonyl carnitine	↓	↑^b^	↑^b^	↑^b^	↑^b^	Fatty acid metabolism	Urine
13	3.15	242.0115	Bicine	↓	↑^b^	↑^b^	↑^b^	↑^b^		Urine
14	2.45	230.0109	Homocysteine thiolactone	↓	↑^b^	↑^b^	↑^b^	↑^b^	Amino acid biosynthesis	Urine
15	1.02	191.0173	Citric acid^d^	↓	↑^b^	↑^b^	–	–	Citrate cycle (TCA cycle)	Urine
16	8.16	268.0634	Isoval eryglutamic acid	↓	↑^b^	↑^b^	–	–		Urine
17	0.76	191.0183	Glucaric acid^d^	↓	↑^b^	↑^b^	–	–	Glyoxylate and dicarboxylate metabolism	Urine
18	14.35	408.1657	9′-carboxy-gama-tocotrienol	↓	↑^b^	↑^b^	–	–	Glyoxylate and dicarboxylate metabolism	Urine
19	7.08	338.0893	Topiramate	↓	–	↑^b^	–	–		Urine
20	13.92	343.0847	Xanthosine	↓	↑^b^	↑^b^	–	–	Purine metabolism	Urine
21	4.51	212.0017	Indoxyl sulfate	↑	↓^c^	↓^c^	↓^c^	↓^c^	Tryptophan metabolism	Urine

a. P-values were calculated from two-tailed Mann–Whitney U-test with a threshold of 0.05. b↑, content increased and *p* < 0.05; c↓, content decreased and *p* < 0.05. d. Confirmed by standard samples.
