# Corrigendum: Frankincense and myrrh suppress inflammation via regulation of the metabolic profiling and the MAPK signaling pathway

**DOI:** 10.1038/srep15597

**Published:** 2015-10-29

**Authors:** Shulan Su, Jinao Duan, Ting Chen, Xiaochen Huang, Erxin Shang, Li Yu, Kaifeng Wei, Yue Zhu, Jianming Guo, Sheng Guo, Pei Liu, Dawei Qian, Yuping Tang

Scientific Reports 5: Article number: 13668; 10.1038/srep13668published online: 09022015; updated: 10292015

In the original version of this Article, the Abstract contained typographical errors.

“Frankincense and myrrh are highly effective in treatment of inflammatary diseases, but lacking of the therapy mechanisms. We undertook this stuty to evaluate the effects on Adjuvant-induced Arthritis (AIA) rats and to explore the underlying mechanisms by analyzing the metabolic profiling and signaling pathway evaluated by expression of inflammatory cytokines, *c-jun* and *c-fos* and corresponding phosphorylation levels”.

now reads:

“Frankincense and myrrh are highly effective in treatment of inflammatory diseases, but lacking of the therapy mechanisms. We undertook this study to evaluate the effects on Adjuvant-induced Arthritis (AIA) rats and to explore the underlying mechanisms by analyzing the metabolic profiling and signaling pathway evaluated by expression of inflammatory cytokines, *c-jun* and *c-fos* and corresponding phosphorylation levels”.

“The metbolic profiling of plasma and urine were clearly improved and twenty-one potential biomarkers were identified”.

now reads:

“The metabolic profiling of plasma and urine were clearly improved and twenty-one potential biomarkers were identified”.

These errors have now been corrected in both the PDF and HTML versions of the Article.

