# Supplementary material for: Frankincense and myrrh suppress inflammation via regulation of the metabolic profiling and the MAPK signaling pathway
Source: Sci Rep. 2015 Sep 2;5:13668. doi: 10.1038/srep13668 (PMC4556964; doi:10.1038/srep13668)

Frankincense and myrrh suppress inflammation via regulation of the metabolic profiling and the MAPK signaling pathway

Shulan Su[1,2], Jinao Duan[1,2]*, Ting Chen[1,2], Xiaochen Huang[1,2], Erxin Shang[1,2], Li Yu[1,2], Kaifeng Wei[3], Yue Zhu[1,2], Jianming Guo[1,2], Sheng Guo[1,2], Pei Liu[1,2], Dawei Qian[1,2], Yuping Tang[1,2]

[1] *Jiangsu Collaborative Innovation Center of Chinese Medicinal Resources Industrialization, National and Local Collaborative Engineering Center of Chinese Medicinal Resources Industrialization and Formulae Innovative Medicine; Nanjing University of Chinese Medicine, Nanjing 210023, PR China*

[2] *Jiangsu Key Laboratory for High Technology Research of TCM Formulae, Nanjing University of Chinese Medicine, Nanjing 210023, PR China*

[3] *Basic Medical College, Nanjing University of Chinese Medicine, Nanjing 210023, PR China*

*Corresponding author.

Prof. Jin-ao Duan

Jiangsu Key Laboratory for High Technology Research of TCM Formulae, Nanjing University of Chinese Medicine, Nanjing 210023, PR China

Tel. / fax: +86 25 85811116

E-mail: duanja@163.com

Supplementary information for the Text S1, Figure S1 and Table S1.

Text S1

Materials and Methods

UPLC – QTOF/MS and UPLC-QqQ/MS analysis. Chromatography was performed on an AcQuity™ UHPLC system (Waters Corp., Milford, MA, USA) with a conditioned auto-sampler at 4 °C. The separation was carried out on an AcQuity UHPLC™ BEH C_{18} column (50 mm × 2.1 mm i.d., 1.7 μm; Waters Corp., Milford, MA, USA), which was maintained at 30 °C. The mobile phase consisted of 0.1% formic acid (HOOCH) in water as solvent A and acetonitrile (ACN) as solvent B. The gradient conditions of the mobile phase were as follows (ES^-): 0~2 min, B: 10%~20%; 2~5 min, B: 55%; and 5~20 min, B: 95%. The gradient conditions of the mobile phase were as follows (ES^+): 0~2 min, A: 10%~20%; 2~5 min, A: 55%; and 5~16 min, A: 90%; 16~20 min, A: 100%. The flow rate was 0.40 mL min^{-1}, and the sample injection volume was 1 μL. Mass spectrometric detection was carried out on an AcQuity Synapt™ mass spectrometer equipped with an electrospray ionization (ESI) interface (Waters, Milford, MA, USA). The ESI source was operated in negative and positive ionization modes. The ionization source conditions were as follows: capillary voltage of 3.5 kV, source temperature of 120 °C and desolvation temperature of 350 °C. The sampling cone voltage was set to 35 V, the extraction cone voltage was 2.0 V (for plasma sample) or 0.7 V (for urine sample), the trap collision energy was 6.0 V, the transfer collision energy was 4.0 V, the trap gas flow was 1.50 mL min^{-1}, and the ion energy was at 1.0 V. Nitrogen and argon were used as cone and collision gases,

respectively. The cone and desolvation gas flow were 50 and 600 L h^{-1}, respectively. The scan time was 0.5 s (for plasma sample) or 0.2 s (for urine sample), and an interval scan time of 0.02 s was used throughout, with a collision energy of 6 eV. The mass spectrometric data were collected from *m/z* 100 to 1000 in centroid mode. Leucine-enkephalin was used as the lock mass, generating an $[M+H]^+$ ion (*m/z* 556.2771) and an $[M-H]^-$ ion (*m/z* 554.2615) at a concentration of 200 pg mL^{-1} and a flow rate of 100 μL min^{-1}. Dynamic range enhancement was applied throughout the MS experiment to ensure that accurate mass measurements were obtained over a wider dynamic range.

BCA protein assay in rat tissues. The BCA protein assay kit was obtained from Westang Bio-tech Co., LTD (Shanghai, China). Test protocol: 1) for preparing the BCA working reagent (WR), mix 50 parts of BCA reagent A with 1 part of BCA reagent B (50:1). The WR is stable for at least 24h when stored at room temperature. 2) Dissolve the standard protein at a concentration of 0.5 mg/mL, preferably in the same diluent as the test samples, otherwise for convenience in 0.9% saline or PBS. 3) Pipette 0, 1, 2, 4, 8, 12, 16 and 20 μL standard protein solutions into 96-microwell plate wells and add diluent into the wells to bring the volume to 20 μL. 4) Pipette 20 μL test sample solution into the wells. 5) Add 200 μL of the WR to each well, mix well. 6) Incubate the plate at 37 °C for 30 minutes. (Note: room temperature for 2h, or 60 °C for 30 minutes. Color reaction increases when the incubation temperature elevated. Increasing the incubation time or temperature increases the net absorbance (A_{562}) measurement, so if the sample concentration is low, increasing incubation time

or temperature could be considered. 6) Measure the absorbance (562nm) of all the samples. Wavelengths from 540-590 nm have also been used successfully. Standard curve fitting algorithms was used to determine the protein concentration for each sample.

Determination of proinflammatory cytokines (IL-2, TNF-*α*, and PGE_2). The assay of IL-2, TNF-*α* and PGE_2 employed the quantitative sandwich enzyme immunoassay technique. Specific antibodies were pre-coated onto the microplate. Standards, control, and samples were pipetted into the wells and the rat cytokine was bound by the immobilized antibody. After washing away any unbound substances, an enzyme-linked antibody specific for rat TNF-*α* or IL-6 was added to the wells. Following color development, the assay was stopped, and the absorbance was read at 450 nm using Biochrom Asys 96 microplate reader, UK. The intensity of the color is proportional to the amount of rat TNF-α or IL-6 bound in the initial step.

The assay of PGE_2 was based on the forward sequential competitive binding technique in which PGE_2 present in the sample competes with a fixed amount of horseradish peroxidase (HRP)-labeled with the corresponding metabolite for sites on specific antibodies precoated onto the wells of the plate. Following incubation and washing, the substrate solution was added to the wells to determine the bound enzyme activity. Following color development, the assay is stopped, and the absorbance is read at 450 nm using Biochrom Asys 96 microplate reader, UK. The intensity of the color is inversely proportional to the concentration of PGE_2 in the samples.

Nitric oxide (NO) determination. Sample preparation: plasma and tissue supernatant

were obtained as described as above and put into 8 tubes with the sample volume of 100 μL respectively. Eight tubes of 100 μL 100 μmol/L KNO_3 were taken as standard and 100 μL ddH_2O as blank. Add 400 μL mixed reagent (nitrate reductase), gently vortex and incubate at 37 °C for 1h. Add reagent 3 and 4 respectively to each tube. Vortex and mix well for 30 second. Keep at room temperature for 40min and then obtained the supernatant by centrifuge. Add reagent and develop the color for 60min. Read OD value at 540nm using a plate reader. 1) Equation of NO in plasma:

$$NO(\mu mol/L) = \frac{SA(OD) - B(OD)}{ST(OD) - B(OD)} \times ST(100\mu mol/L) \times D)$$

SA: Sample, ST: Standard, B: Blank, D: Dilution factor

2) Equation of NO in tissue:

$$NO(\mu mol/gprot) = (\frac{SA(OD) - B(OD)}{ST(OD) - B(OD)} \times ST(20\mu mol/L) \times D)/Sp)$$

SA: Sample, ST: Standard, B: Blank, D: Dilution factor, Sp: Sample protein (gprot/L)

Malondialdehyde (MDA) assay. Sample solutions were put into 8 tubes with the volume of 50 μL and separate 8 tubes for blank and standard, respectively. Add mixed reagent into each tube. Vortex and mix. Incubate at 95 °C for 40 min. After cooling, centrifuge at 3500-4000 rpm for 10 min. Pipette 200 μL from each tube into a 96 well plate for analysis. Measure the absorbance at 532 nm (A_{532}). Calculate the amount of MDA with equations as below: 1) Equation of MDA assay in serum:

$$MDA(nmol/mL) = \frac{SA(OD) - B(OD)}{ST(OD) - B(OD)} \times ST(10nmol/mL) \times D)$$

SA: Sample, ST: Standard, B: Blank, D: Dilution factor

2) Equation of MDA assay in tissue:

$$MDA(nmol/mgprot) = (\frac{SA(OD) - B(OD)}{ST(OD) - B(OD)} \times ST(10nmol/mL) \times D))/Sp$$

SA: Sample, ST: Standard, B: Blank, D: Dilution factor, Sp: Sample protein (gprot/L)

Metabolomic data processing and multivariate analysis. UPLC/MS data were detected and noise-reduced in both the UPLC and MS domains such that only true analytical peaks were selected for further processing by the software. A list of the peak intensities detected was then generated for the first chromatogram using the Rt-*m/z* data pairs as identifiers. The resulting normalized peak intensities form a single matrix, with Rt-*m/z* pairs for each file in the dataset. All processed data from each chromatogram were normalized and Pareto-scaled prior to the multivariate statistical analysis.

All data from the plasma and urine samples were processed using the MarkerLynx application manager for MassLynx 4.1 and MarkerLynx software (Waters Corp., Milford, USA). The intensity of each ion was normalized with respect to the total ion count to generate a data matrix consisting of the retention time, *m/z* value, and normalized peak area. The multivariate data matrix was analyzed using *EZ*info software (Waters Corp., Milford, USA). Unsupervised segregation was examined with a principal components analysis (PCA) using Pareto-scaled data. A partial least squared discriminant analysis (PLS-DA) and an orthogonal partial least-squared discriminant analysis (OPLS-DA) were used to identify the various metabolites responsible for the separation between the model and normal groups. Potential biomarkers of interest were extracted from the S-plots that were constructed following

the OPLS-DA analysis, and the biomarkers were chosen based on their contribution to the variation and correlation within the dataset.

An internal 5-fold cross-validation was carried out to estimate the performance of the PLS-DA models. The calculated $R^2Y_{(cum)}$ estimates how well the model represents the fraction of explained Y-variation, and $Q^2_{(cum)}$ estimates the predictive ability of the model. Models are considered excellent when the cumulative values of R^2Y and Q^2 are greater than 0.8. In addition to cross-validation, 200 permutation tests were also performed to validate the model. The VIP (variable importance in the projection) value is a weighted sum of squares of the PLS weights that reflects the relative contribution of each X variable to the model. The variables with VIP > 1 were considered to be influential for sample separation in the score plots generated from PLS-DA analysis [26]. Ultimately, different metabolic features associated with the model group and the SFZYD treatment group were obtained based on cutoff points of both VIP values from a 5-fold cross-validated PLS-DA model and critical *p*-values from a univariate analysis. In addition, the corresponding fold-change was calculated to show the degree of variation in metabolite levels between groups.

Biomarker identification and metabolic pathway analysis. This information was then used to search multiple databases, including ChemSpider database (www.chemspider.com), PubChem (http://ncbi.nim.nih.gov/), and MetFrag (http://msbi.ipb-halle.de/MetFrag/). The metabolic pathway analysis of potential biomarkers was performed using MetPA (http://metpa.metabolomics.ca./MetPA/faces/Home.jsp) based on the KEGG (http://www. genome.jp/kegg/), Human Metabolome Database

(http://www.hmdb.ca/), and METLIN (http://metlin.scripps.edu/). Potential biological roles were evaluated by an enrichment analysis using MetaboAnalyst.

Figure S1

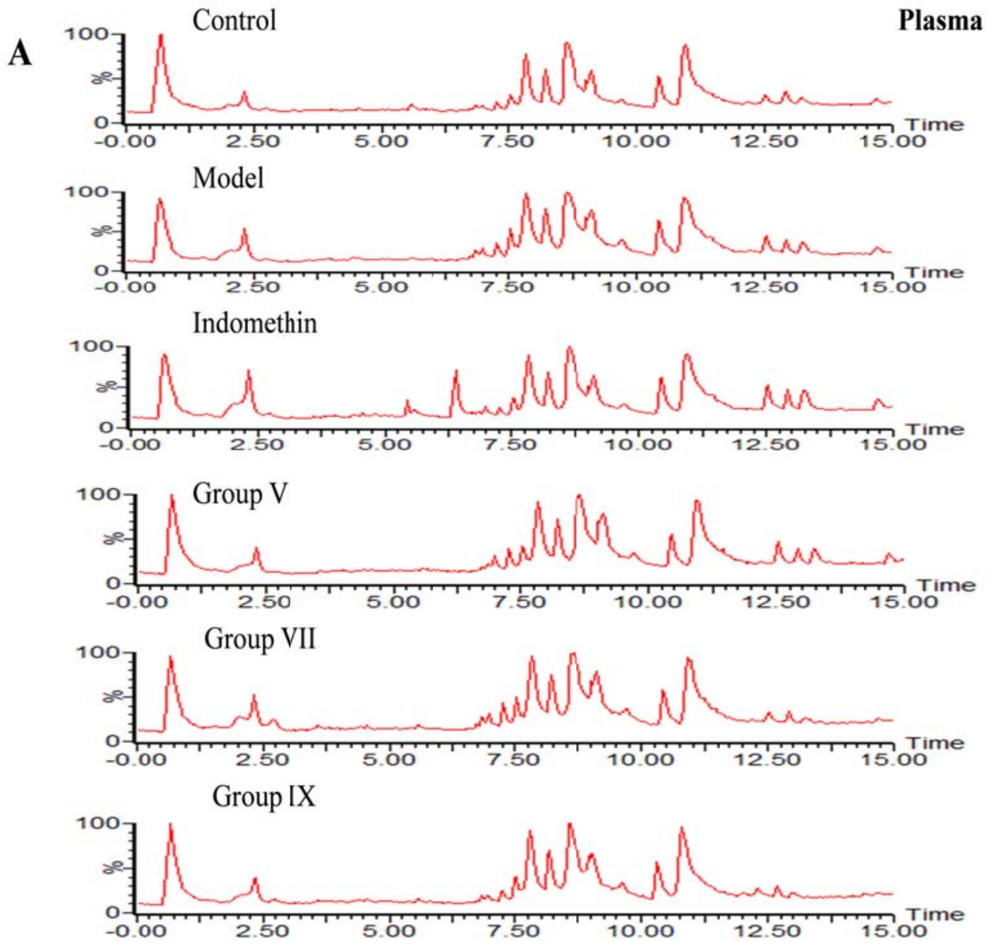

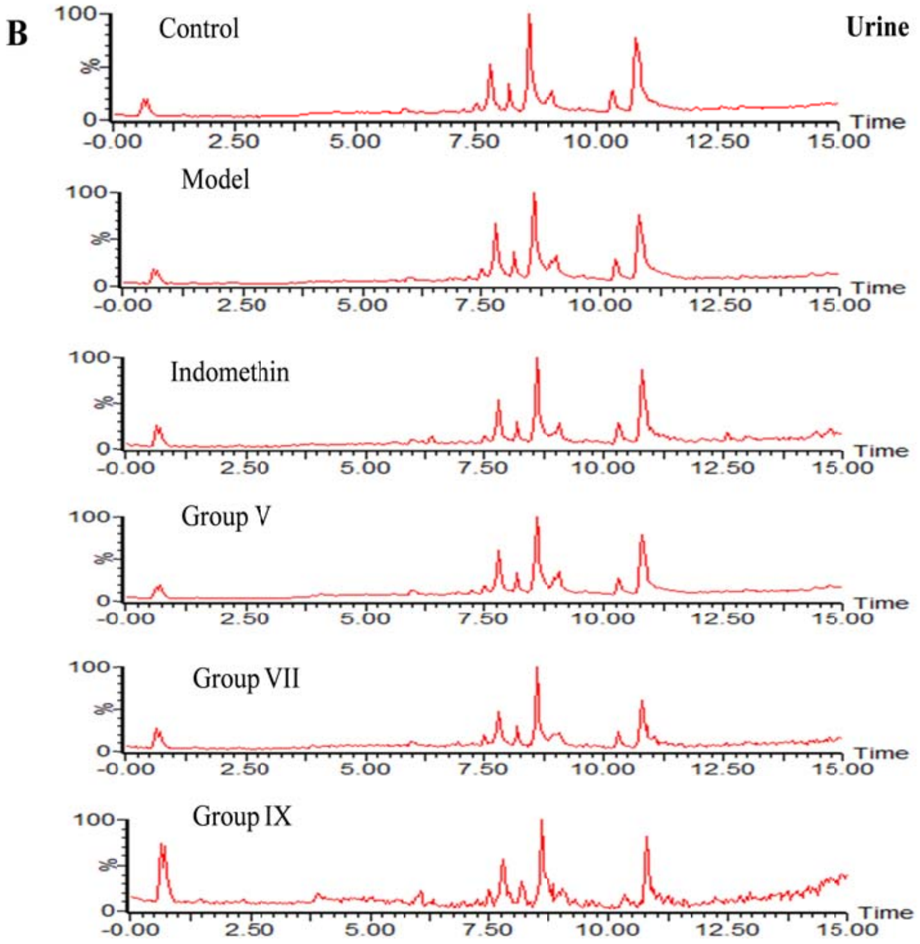

Figure S1 The total ion chromatograms of rat plasma (A) rat urine (B)

Table S1

Table S1 The optical density (OD) of Control group and five compounds groups by MTT

Groups	Dosage(μg/ml)	OD (mean±SD)
Control group	0.00	0.663 ±0.039
Compund 1	15.00	0.670 ±0.036
	35.00	0.655 ±0.017
	70.00	0.657 ±0.035
	140.00	0.610 ±0.023
	280.00	0.582 ±0.022
Compund 2	0.25	0.678 ±0.036
	0.50	0.633 ±0.011
	1.00	0.604 ±0.038
	2.00	0.581 ±0.012
	4.00	0.532 ±0.010
Compund 3	0.25	0.688 ±0.004

	0.50	0.674 ±0.031
	1.00	0.641 ±0.022
	2.00	0.601 ±0.029
	4.00	0.579 ±0.017
Compund 4	1.00	0.680 ±0.023
	2.00	0.675 ±0.007
	4.00	0.615 ±0.016
	8.00	0.567 ±0.013
	16.00	0.526 ±0.041
Compund 5	0.60	0.678±0.026
	1.15	0.660 ±0.027
	2.30	0.644 ±0.032
	4.60	0.588 ±0.012
	9.20	0.570 ±0.019

Wester blots

3 hr

ERK

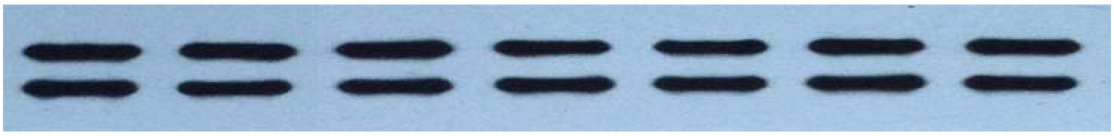

JNK

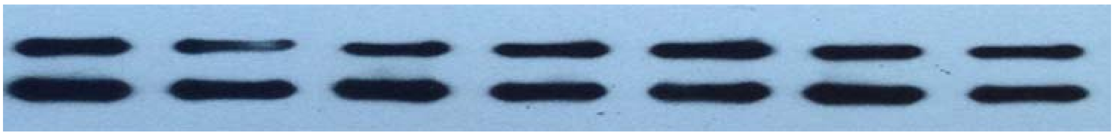

p38

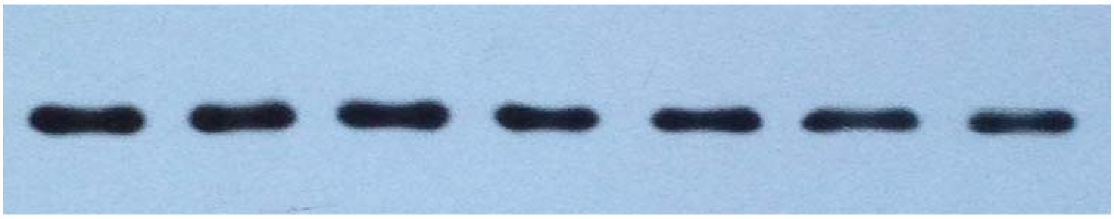

p-ERK

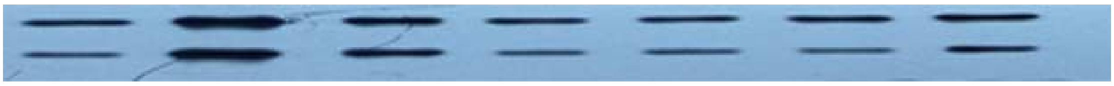

p-JNK

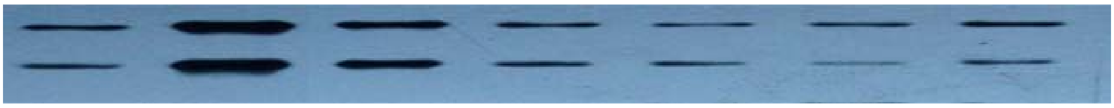

p-P38

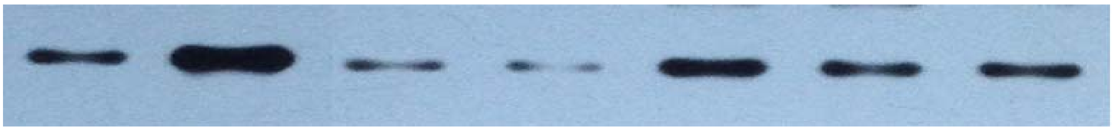

6 hr

ERK

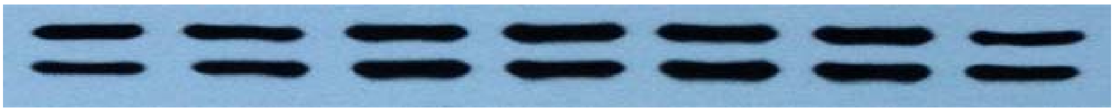

JNK

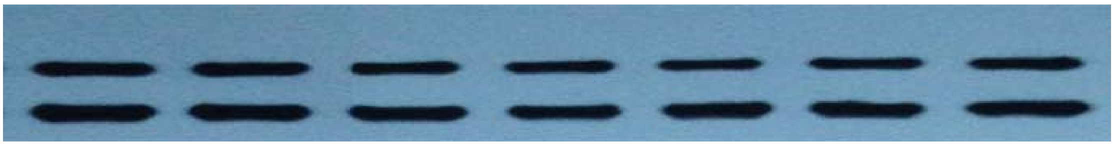

P38

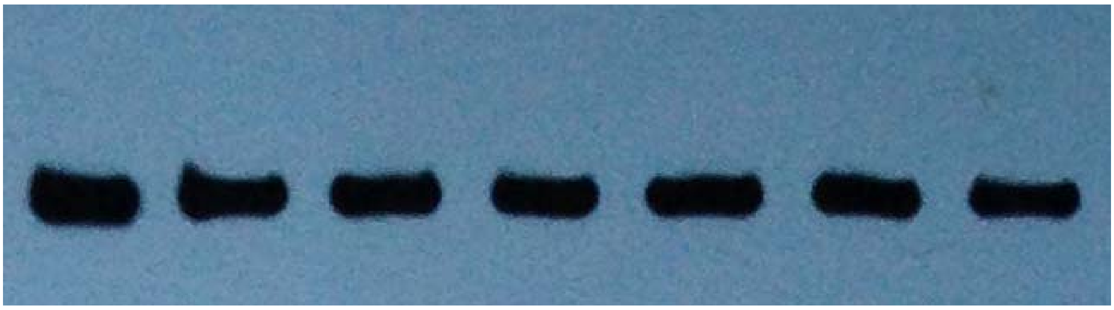

p-ERK

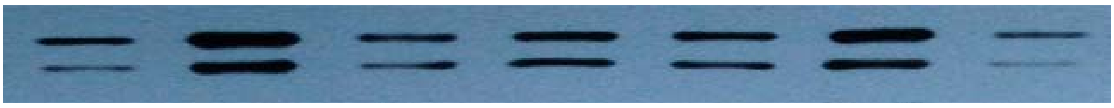

p-JNK

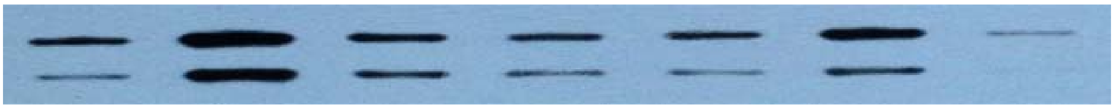

p-P38

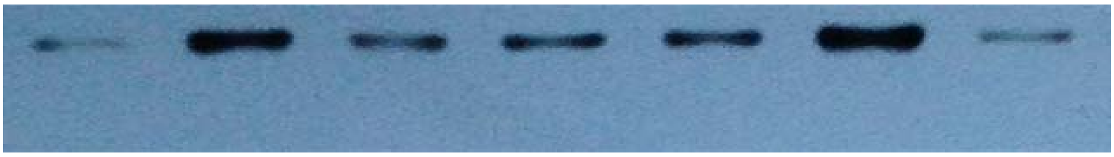

Supplement: Supplementary Information [file srep13668-s1.pdf]
